# Compositionally Aware Phylogenetic Beta-Diversity Measures Better Resolve Microbiomes Associated with Phenotype

**DOI:** 10.1128/msystems.00050-22

**Published:** 2022-04-28

**Authors:** Cameron Martino, Daniel McDonald, Kalen Cantrell, Amanda Hazel Dilmore, Yoshiki Vázquez-Baeza, Liat Shenhav, Justin P. Shaffer, Gibraan Rahman, George Armstrong, Celeste Allaband, Se Jin Song, Rob Knight

**Affiliations:** a Department of Pediatrics, University of California San Diego School of Medicine, La Jolla, California, USA; b Bioinformatics and Systems Biology Program, University of California, San Diego, La Jolla, California, USA; c Center for Microbiome Innovation, University of California, San Diego, La Jolla, California, USA; d Jacobs School of Engineering, University of California San Diego, La Jolla, California, USA; e Biomedical Sciences Program, University of California, San Diego, La Jolla, California, USA; f Center For Studies in Physics and Biology, Rockefeller University, New York, New York, USA; g Department of Bioengineering, University of California, San Diego. La Jolla, California, USA; h Department of Computer Science and Engineering, University of California, San Diego, La Jolla, California, USA; University of Massachusetts Medical School

**Keywords:** beta-diversity, phylogenetics, compositional data analysis

## Abstract

Microbiome data have several specific characteristics (sparsity and compositionality) that introduce challenges in data analysis. The integration of prior information regarding the data structure, such as phylogenetic structure and repeated-measure study designs, into analysis, is an effective approach for revealing robust patterns in microbiome data. Past methods have addressed some but not all of these challenges and features: for example, robust principal-component analysis (RPCA) addresses sparsity and compositionality; compositional tensor factorization (CTF) addresses sparsity, compositionality, and repeated measure study designs; and UniFrac incorporates phylogenetic information. Here we introduce a strategy of incorporating phylogenetic information into RPCA and CTF. The resulting methods, phylo-RPCA, and phylo-CTF, provide substantial improvements over state-of-the-art methods in terms of discriminatory power of underlying clustering ranging from the mode of delivery to adult human lifestyle. We demonstrate quantitatively that the addition of phylogenetic information improves effect size and classification accuracy in both data-driven simulated data and real microbiome data.

**IMPORTANCE** Microbiome data analysis can be difficult because of particular data features, some unavoidable and some due to technical limitations of DNA sequencing instruments. The first step in many analyses that ultimately reveals patterns of similarities and differences among sets of samples (e.g., separating samples from sick and healthy people or samples from seawater versus soil) is calculating the difference between each pair of samples. We introduce two new methods to calculate these differences that combine features of past methods, specifically being able to take into account the principles that most types of microbes are not in most samples (sparsity), that abundances are relative rather than absolute (compositionality), and that all microbes have a shared evolutionary history (phylogeny). We show using simulated and real data that our new methods provide improved classification accuracy of ordinal sample clusters and increased effect size between sample groups on beta-diversity distances.

## INTRODUCTION

In recent decades, microbial sequencing data have been analyzed by a growing community of scientists to address a wide range of topics from human health to environmental monitoring. However, such data have specific properties that make proper analysis using conventional methods challenging. Specifically, microbial sequencing data are highly sparse (very few species/genes shared between samples), nonnormally distributed, and compositional in nature (1–3).

The comparison of microbiome sequencing data among samples is commonly performed through dimensionality reduction on a distance matrix that represents the beta-diversity between each pair of samples. There are many different metrics that quantify beta-diversity, each of which attempts to overcome a unique challenging characteristic of microbiome sequencing data. For example, methods such as Bray-Curtis (4) and Jaccard (5) produce similarities that are quantitative and qualitative, respectively. Although these methods are simple, essentially operating off the overlap in set membership (Jaccard) or weighted membership (Bray Curtis), their equations make particular assumptions of the data being examined, which can produce nuisance similarities in the context of microbiome data and artifacts in downstream steps such as dimensionality reduction (6). Briefly, these assumptions include the following: all organisms are equally related, the data are noncompositional, the data are dense, the data require rarefaction (or some method to account for variation in sampling effort), and samples are independent.

Using UniFrac distances for estimating beta-diversity integrates phylogenetic information, which overcomes the assumption that all species are equally related and greatly improves the ability to discriminate between sample groups (7, 8). However, the UniFrac variant that utilizes weighted membership requires rarefaction, assumes dense data, and does not account for the compositional nature of the data. Weighted membership methods such as Aitchison distance utilize the centered log-ratio transformation (CLR) to account for the compositional nature of the data and have been adapted to incorporate phylogenetic information (i.e., Ratio and Information UniFrac) (9, 10). These metrics still assume the data are dense and require the imputation of missing values, often through the addition of a pseudocount. Robust principal-component analysis (RPCA), builds upon the ideas of Aitchison PCA, but instead treats all unobserved values as missing through an adaptation of the CLR that is robust to missing data (RCLR) (11). RPCA has also been adapted to account for repeated-measure study designs through Compositional Tensor Factorization (CTF) (12). However, both RPCA and CTF fall short in the assumption that all organisms are equally related. In total, each of these metrics addresses different combinations of challenges posed by microbiome data, often yielding varying results and convoluting the field (Table S1 in the supplemental material).

Here, we propose an extension to RPCA and CTF, called phylogenetic-RPCA and -CTF (phylo-RPCA -CTF), that accounts for the evolutionary relationships among the microbes present within a sample. This is accomplished through a postorder transformation of a feature table, a data layer that underpins the classic Fast UniFrac (13) algorithm, combined with the RCLR transformation that underpins both RPCA and CTF. This yields a dimensionality reduction and beta-diversity metric that explicitly accounts for the relationships among features in addition to the sparsity and compositional nature of the data.

## RESULTS

### Description of phylogenetic RPCA.

**FIG 1:**
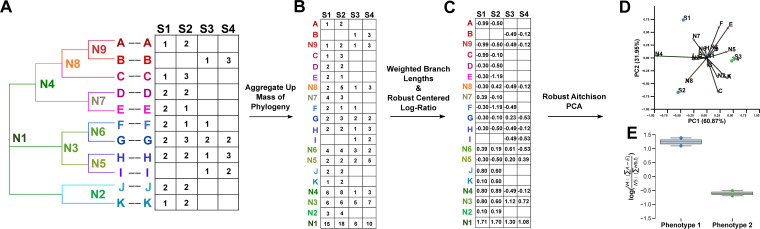
Overview of the algorithm underlying phylo-RPCA and phylo-CTF. The input of a table of count data and a phylogeny representing the features of the table (A). First, the table is expanded to represent all nodes up to the root of the phylogeny through summing up each node (B), second, the closure of the expanded table is multiplied by the branch lengths following Hamady 2010 (13), and the data is then transformed with the rclr (C) and then RPCA is performed. The output provides a phylogenetic biplot where arrows are both leaves and internal nodes of the input phylogeny (D) whose direction can inform log-ratios of aggregated leaves counts (E).

In order to integrate a community’s phylogeny into the RCLR transformation and therefore into RPCA and CTF, we borrow the count arrays from the Fast UniFrac algorithm (13). First, we are given a table of count data where each feature (i.e., microbe, ASV, gene) in the table corresponds to tips in a phylogenetic tree (Fig. 1A). Second, following the methodology of Fast UniFrac, all internal nodes are exposed in the table by aggregating the descendants under each node in the phylogeny (Fig. 1B). Third, the aggregated table is closed and the branch lengths of each node and tip in the tree are multiplied. Missing values are treated as missing and the robust centered log-ratio transformation is applied only to the observed values (Fig. 1C). In the case of cross-sectional study designs, RPCA can be applied, and in the case of repeated measures studies, CTF. After dimensionality reduction through RPCA, the data can be viewed as a compositional biplot where the arrows represent the feature loadings along a principal component axis, which include both tips and internal nodes of the tree (14). These loadings are then used to identify key features that contribute to the ability to discriminate between sample groups. Subsequently, we use these features as the numerator and denominator in a log-ratio. In this case, the numerator and denominator correspond to the sum of counts across all the tips lower in the hierarchy (Fig. 1D and E). Moreover, the log-ratio of zero is undefined; therefore, log-ratios of sparse microbiome data often rely on an aggregation of many features (15). The log-ratio of the sum of tips under two internal nodes provides an intuitive solution to provide a dense ratio and prevent sample drop-out from missing or imputed zero values.

### Simulations.

To benchmark the impact of phylogenetic weighting for RPCA, we created data-driven simulations based on microbiome samples from the Earth Microbiome Project 500 (EMP500). We simulated a shotgun metagenomics data set based on animal, saline, and nonsaline environments (16) (Fig. S1) (see Materials and Methods for details). Data-driven simulations were chosen as a proof-of-concept to see how both sequencing depth and the proportion to which a phylogeny can impact phylo-RPCA.

**FIG 2:**
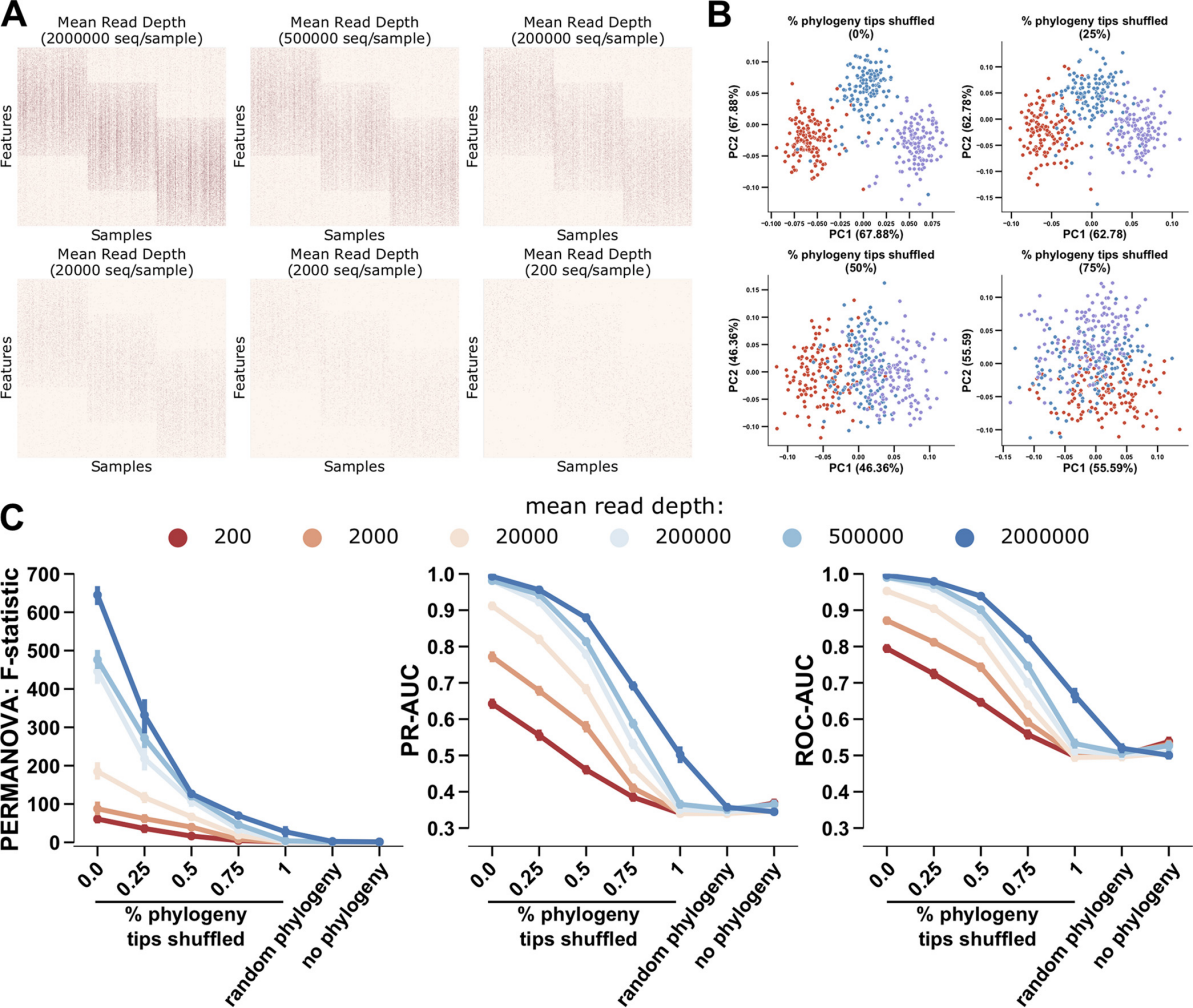
As phylogeny becomes more synchronized with the samples’ clusters, the additional benefit of phylogenetic information in RPCA increases. A data-driven simulation of shotgun microbiome data of three sample groups, based on EMP500 data, with reduced sequencing depth across plots from 2,000,000 to 200 reads (A). Comparison of phylogenetic RPCA sample clustering with a randomly generated tree and as a percentage of the tips of the phylogenetic tree, originally perfectly representing the features clustering the samples, are randomly shuffled 10-fold (B). Comparison across simulation read depth (colors from low to high) and phylogenetic-feature-sample cluster synchrony (*x* axis) for PERMANOVA F-statistic (left), area under the precision-recall curve (PR-AUC, middle), and area under the receiver operator characteristic curve (right) (C).

The simulated data were generated such that the majority of microbial features (e.g., ASV or genome) are most abundant in one of three sample groups (i.e., animal, saline, and nonsaline environments), with an additional subset of features shared between two or all groups. The representative phylogeny, taken from the EMP500 data set, was artificially sorted such that the postorder traversal of the tips match the order of the sample clusters. Next, we generated data ranging from 200 to 2 million sequences per sample in addition to desynchronizing the level of association between the phylogenetic information and sample clusters by randomly sorting 0%, 25%, 75%, or 100% of the tip IDs of the phylogenetic tree 10 times. In order to produce a comparison with no possible phylogenetic information retained, a random phylogeny containing the tip IDs of the original tree was produced for comparison. For each simulation, we ran phylo-RPCA as well as RPCA without any phylogenetic information (Fig. 2A). Of note, the greater the percentage of phylogeny tip IDs that were randomly shuffled, the less the three sample groupings separated (Fig. 2B). In order to quantify these observations, output distance matrices representing beta-diversity were compared via permutational multivariate analysis of variance (PERMANOVA) pseudo-F-statistic, and ordinations via supervised k-nearest neighbor (KNN) classification cross-validation (50:50 split) evaluated through the area under the precision-recall curve (PR-AUC) and area under the receiver operator characteristic curve (ROC-AUC) (17).

We observed that with perfectly aligned phylogeny and sample clustering, phylo-RPCA provides a 600-fold increase in the F-statistic effect size and a 66% decrease in the PR-AUC and ROC-AUC. A decrease in sequencing depth led to a 10-fold decrease in the F-statistic and a 36% decrease in the PR-AUC and ROC-AUC. This observation is consistent with previous evaluations of RPCA (11). Similarly, large decreases in the F-statistic, as well as the PR/ROC-AUC, were observed between the fully synchronized and no phylogeny at all. However, in the case of a random phylogeny, RPCA and phylo-RPCA are similar in performance (Fig. 2C). This demonstrated a proof-of-concept that through disrupting the phylogeny, some phylogenetic signal is better than none and that even poorly constructed or representative phylogenies provide some benefit.

### Case studies.

Next, we compared the discriminatory ability of phylo-RPCA and phylo-CTF to state-of-the-art beta-diversity metrics, using two 16S rRNA gene amplicon sequencing data sets. The first, a cross-sectional data set, compared the skin microbiomes of subjects across a gradient of urbanization in South America, represented by village (*n* subjects, 164) (18). The second, a repeated-measures data set, follows the fecal contents of infants from birth across the first 2 years of life between two birth modes, vaginal or cesarean section (C-section) delivery (*n* subjects, 43 with monthly sampling) (19).

**FIG 3:**
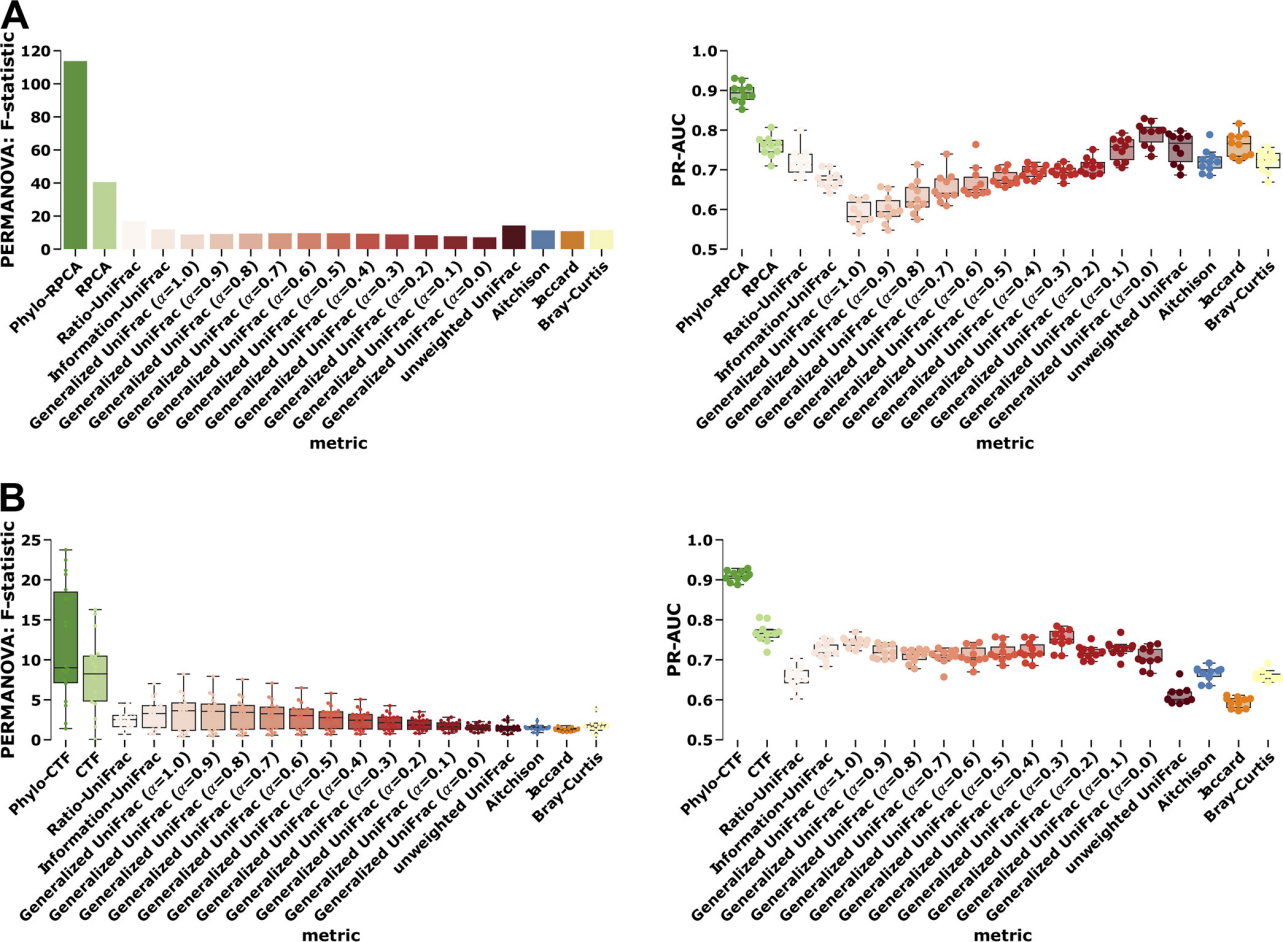
Phylogeny improves discriminatory power in cross-sectional data and in repeated measure data compared to existing methods. Comparison of phylogenetic RPCA/CTF (green) against nonphylogenetic version (light-green), Aitchison PCA (blue), Jaccard (orange), phylogenetically informed unweighted UniFrac, and generalized UniFrac with alpha varying level of abundance weighting (colored in reds from least to most weighted by abundance). Compared by PERMANOVA F-statistic on beta-diversity distances (left column), 10-fold KNN classification cross-validation was evaluated through the area under the precision-recall (right column). Comparison of cross-sectional data by hand skin bacterial communities from McCall et al. compared across villages representing an urbanization gradient from Peru to Brazil (A). Repeated measure comparison of fecal bacterial communities from ECAM data set compared across age and compared by birth mode (B).

Of the many possible beta-diversity metrics, we compared phylo-RPCA and phylo-CTF to a selection of widely-used metrics: Jaccard (5), Bray-Curtis (4), Aitchison (9), Ratio-UniFrac (10), Information-UniFrac (10), and UniFrac ranging in the amount of weighting of abundances from unweighted (20) to weighted through varied alphas of generalized UniFrac (0 to 1 in increments of 0.1 where 0 is similar to unweighted UniFrac and 1 is weighted UniFrac) (21). We also included the nonphylogenetic counterparts RPCA (11) and CTF (12) in the comparison. Following the same regime as the simulation data, each metric was elevated through both PERMANOVA F-statistic and KNN classification cross-validation (50:50 split) evaluated by PR-AUC (ROC-AUC was not compared due to unbalanced sample groups). In both data sets, the best performing metrics were phylo-RPCA and phylo-CTF followed by their nonphylogenetic counterpart (i.e., RPCA and CTF) with a 2-fold improvement in the F-statistic and a 14% improvement in PR-AUC in both cases. Moreover, Ratio-UniFrac outperformed Aitchison, and UniFrac outperformed Jaccard, their respective nonphylogenetically weighted comparable metrics. In total, compared to all other metrics, phylo -RPCA and -CTF provided markedly improved results (Fig. 3A and B).

One major benefit of phylogenetic RPCA and CTF is that all the internal nodes are provided in the feature loadings, providing a guide to the importance of phylogenetic partitions along the principal component axis where samples are also separated. This allows us to rank each internal node in relation to the samples and their phenotypes in the metadata. We provide an interactive plugin to allow this exploration - of a phylogenetic tree and node importance - through a combination of Emperor (22) and Empress (23) called Empire (interactive plots can be explored here and here for the cross-sectional and repeated-measures data respectively). To validate the association observed in the feature/node loadings, log-ratios of all aggregated features/tips below two nodes can be used (see Materials and Methods for more details).

**FIG 4:**
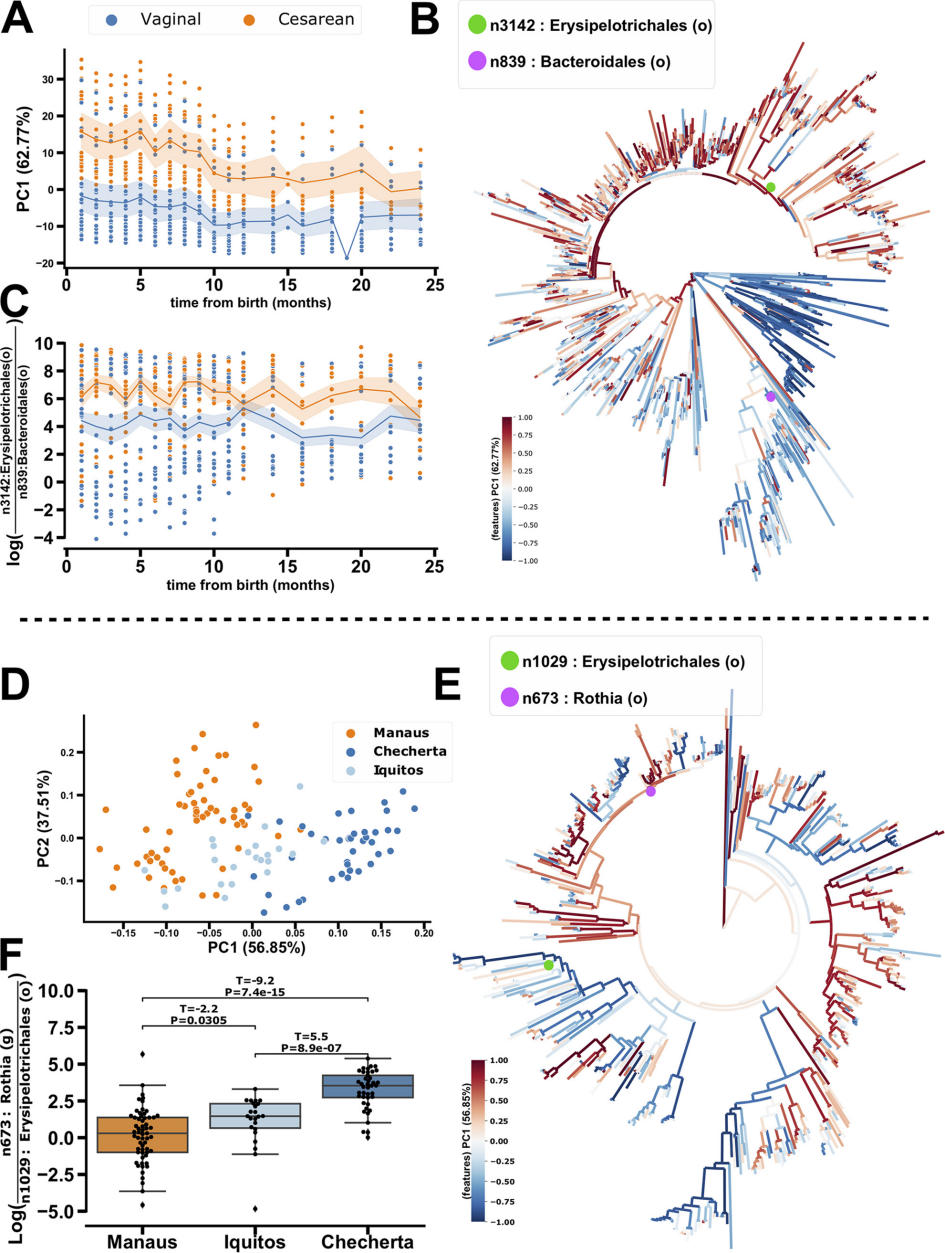
Phylogenetic-RPCA and -CTF resolve ordinal and phylogenetically aggregated log-ratios in birth-mode (top) and westernization gradients by village (bottom) respectively. Phylo-CTF ordination PC1 (*y* axis) colored by birth mode (A), Bacterial and Archaeal phylogeny colored by PC1 feature loadings that also separate the respective sample groups PC1 for phylo-CTF (B), and log-ratio of high (numerator, colored by a purple dot in the phylogeny) and low (denominator, colored by a green dot in the phylogeny) value loadings identified in the respective phylogenies and sample groupings for phylo-CTF (C). Phylo-RPCA PC1 (*x* axis) and PC2 (*y* axis) colored by village across urbanization gradient (D), phylogeny colored by PC1 feature loadings (E), log-ratio of high (numerator, colored by a purple dot in the phylogeny) and low (denominator, colored by a green dot in the phylogeny) value loadings identified in the respective phylogenies and sample groupings for phylo-RPCA (F).

In order to demonstrate this, we first explore the repeated measures data set. The infants who were born by C-section separate from those vaginally born along the first PC axis (Fig. 4A). By coloring the associated phylogeny with the PC1 feature/node loadings from phylo-CTF we can see associations of phylogenetic partitions more associated with C-section or vaginally born infants by larger positive and negative PC1 values in the tree (Fig. 4B). In particular, the log-ratio of the positively loaded C-section-associated internal node n3142 (lowest common ancestor, order Erysipelotrichales) and negatively loaded vaginally-associated node n839 (lowest common ancestor, order Bacteroidales) in the numerator and denominator, respectively, recapitulates the separation by birth mode seen in the ordination (Fig. 4C). The order Bacteroidales has been previously observed in a higher abundance in vaginally born infants compared to those born by C-section (24).

This same process can be applied to the cross-sectional data. For example, the position in the ordination where PC1 separates by village and the degree of urbanization (Fig. 4D) can be projected onto the phylogeny to identify key phylogenetic partitions (Fig. 4E). In particular, the ratio of the highly loaded node n673 (lowest common ancestor, order Erysipelotrichales) to n1029 (lowest common ancestor, genus Rothia) significantly separates the villages in the same direction as the ordination (Fig. 4F). In particular, representation of the order Erysipelotrichales was also significantly increased in the more urbanized villages relative to *Rothia*. In this way, phylo-RPCA and -CTF can be used to identify evolutionary breakpoints, presented in log-ratios of highly loaded internal nodes of the phylogeny, that help explain the separations observed in the ordinations.

## DISCUSSION

Here we demonstrated that there is an additive improvement in estimating beta-diversity and performing dimensionality reductions on microbiome sequencing data by explicitly accounting for the evolutionary relationships among microbes, sparsity, and the compositional nature of the data. We showed through simulations that phylogenetic tree integration improves, and in the worst case does not hinder, the ability to compare microbial communities between samples. In addition, phylo-RPCA and -CTF quantitatively improved the ability to discriminate between sample groups compared to their nonphylogenetic counterparts and techniques for estimating beta-diversity commonly used in the field.

Importantly, because the phylo-RPCA/CTF provides internal node detail that is linked to sample information, one can identify groups of features based on phylogenetic partitions that are associated with sample clusters. These phylogenetically grouped features provide a more precise alternative to log-ratios of taxonomic groups. In either case of aggregated log-ratios, it is critical to prevent overlapping features between the numerator and denominator sums in the log-ratio, because doing so produces misleading results (25).

While the advances here are important, there are still numerous challenges and considerations when utilizing phylo-RPCA or -CTF. First, the increased feature space dramatically increases the runtime. In the case of large tables (e.g., N features > 10,000), including those used in the case studies, the increased runtime could be prohibitive depending on resources available to the researcher (Table S2). Future work will address this problem; however, one option now is to use the provided methods for a phylogeny-guided pruning of the feature space (see Materials and Methods for more details). Second, both RPCA and CTF algorithms currently require recalculation with new samples, and is an active area of research (26). Third, as described previously, the low-rank assumption of RPCA, CTF, and many other dimensionality reduction methods can be misleading in high-rank data (6, 11, 12). Finally, the CTF algorithm is aware of repeated measures, but does not encode the order of those measures; future work is required to adapt the algorithm to be aware of the order present in longitudinal study designs. Moreover, there are many future directions for incorporating other forms of prior knowledge into these methodologies.

## MATERIALS AND METHODS

### Phylogenetic RPCA and CTF.

Phylogenetic RPCA and CTF assume two inputs being a phylogenetic tree and a matrix of counts where the features of the matrix are all represented in the phylogeny. The phylogenetic tree is denoted as *P*〈ϒ, Λ〉 where the nodes of the tree are ϒ = *v*_1_, *v*_2_, … *v*_a_ and the branch weights *A* = *ϵ*_1_, *ϵ*_2_, …, *ϵ*_b_. The count matrix is denoted as *x_i_*_,_*_j_* with *x*_1_, *x*_2_, …, *x_i_* as the features corresponding to leaves of the tree for each sample *x_j_*.

As we previously published (11), the approximate clr transform only defined on nonzero counts circumvents the problem of partially observed (sparse) data. The robust clr transform is given as

(1) $$\mathrm{rclr}\left(x\right)=\left[\log\frac{x_{1}}{g_{r}\left(x\right)},\ldots ,\log\frac{x_{D}}{g_{r}\left(x\right)}\right]$$

(2) $$g_{r}\left(x\right)={\left(\prod_{i\in \Omega_{x}}^{}x_{i}\right)}^{1/|\Omega_{x}|}$$

where *x_i_* is the abundance of taxa i, Ω*_x_* is the set of observed taxa in sample *x*, and *g_r_*(*x*) is the geometric mean only defined on observed taxa. This can be redefined in total by the following where *y_ij_* is defined only where *x_ij_* > 0.

(3) $$y_{\mathrm{ij}}=\;\log x_{\mathrm{ij}}-\frac{1}{\left|\Omega_{x_{i.}}\right|}\sum_{k\in \Omega_{x_{i.}}}^{}x_{k}-\frac{1}{\left|\Omega_{x_{.j}}\right|}\sum_{i\in \Omega_{x_{.j}}}^{}x_{k}$$

In order to incorporate the phylogenetic weights, we follow from Fast UniFrac first defined in (13). First, we represent each node of the phylogenetic tree in *x_i_*_,_*_j_* by calculating the observed counts of every node up the tree and the counts if its descendants. This gives a matrix of *x_a_*_,_*_j_* where *x_i_*_,_*_j_* with *x*_1_, *x*_2_, …, *x_i_* corresponds to ϒ = *v*_1_, *v*_2_, … *v_a_*. The total weight is defined as the sum of the branch lengths *W*(*ϵ_i_*), which is vectorized and defined as *V_l_*. In Fast UniFrac the distance between sample *x* and *x*′ is given as

$$d_{U}\left(x,x^{\prime }\right)=\frac{\sum_{i=1}^{m}V_{l}\cdot \left(x⊕x^{\prime }\right)}{\sum_{i=1}^{m}V_{l}\cdot \left(x\vee x^{\prime }\right)}$$

We can adapt this methodology to the rclr transformation. The phylogenetic-rclr transform is given by:

(4) $$y_{\mathrm{aj}}=\;\log(V_{l}\cdot x_{\mathrm{aj}})-\frac{1}{\left|\Omega_{V_{l}\cdot x_{a.}}\right|}\sum_{k\in \Omega_{V_{l}\cdot x_{a.}}}^{}x_{k}-\frac{1}{\left|\Omega_{x_{.j}}\right|}\sum_{a\in \Omega_{x_{.j}}}^{}x_{k}$$

Beta-diversity calculation and dimensionality reduction of the phylogenetic-rclr transformed values, are performed through the same methodology as introduced in the original RPCA and CTF algorithms for cross-sectional and repeated measure study designs, respectively (11, 12).

### Simulation benchmarks.

Data-driven simulations were used to benchmark characteristics of the data while making the fewest assumptions of the microbial distributions as possible. We utilized a previously published procedure introduced in the original RPCA and CTF manuscripts (11, 12). The EMP500 data set was chosen due to the large range in sequencing depths, environments sampled, and distinct three clusters (animal, saline, and nonsaline environments) (16). The software used to generate the simulations is available at https://github.com/gibsramen/BIRDMAn_Jr. Briefly, a microbial proportion table was drawn in three blocks, replicating the EMP500 data, through the following distributions (25):

(5) $$x_{\mathrm{ij}}=\frac{1}{\sqrt{2\pi \sigma^{2}}}e\mathrm{xp}\left(\frac{{\left(\mu_{i}-g_{j}\right)}^{2}}{2\sigma^{2}}\right)$$

(6) $$p_{\mathrm{ij}}=\frac{x_{\mathrm{ij}}}{\Sigma_{k}x_{\mathrm{kj}}}$$

The *p_ij_* were induced with both normally and randomly generated noise. In order to simulate the final subsampled count table *y_ij_*, a Poisson-log normal (PLN) distribution was applied given by

(7) $$\lambda_{\mathrm{ij}}=np_{\mathrm{ij}}$$

(8) $$y_{\mathrm{ij}}=\mathrm{PLN}\left(\lambda_{\mathrm{ij}},\phi \right)$$

The parameters of the simulation were optimized to replicate the EMP500 data set. Moreover, the EMP500 phylogenetic tree was post order sorted and the tip IDs were assigned to the features in the order by which they grouped into each simulated block. Next, sequencing depth was simulated from 200 to 2 million reads/sample. At each sequencing depth, the phylogenetic tree IDs were shuffled at a proportion of 0, 25, 75, and 100%. A randomly generated phylogenetic tree was produced through ngesh (v. 1.1.1) on fast mode using otherwise default parameters with the original phylogenetic tree tip IDs as input (27). This procedure was repeated 10 times. Each simulation was then processed with phylogenetic-RPCA or RPCA and compared through PERMANOVA (17) F-statistic or KNN classification on the beta-diversity distances and ordinations respectively. To assess the classification accuracy, KNN classification was performed with 10-fold 50:60 cross-validation evaluating area under curve and average precision-recall (APR) prediction accuracy at each fold iteration via scikit-learn (v.0.21.2) (28).

### Case studies.

The two real data sets were acquired from and processed through the default Qiita analysis. The skin urbanization study (18) was filtered to retain features greater than 10 total counts across all samples, and the ECAM data (19) were filtered for singletons. Each data set was rarefied for noncompositional metrics through QIIME2 (v.2021.2) (29) to retain at a minimum 75% of the samples, which was 11939 and 29420 for ECAM and the skin data set, respectively. For each data set Jaccard, Bray–Curtis, Weighted UniFrac, Unweighted UniFrac, Aitchison, RPCA, and CTF distances were calculated through QIIME2 (v.2019.7). Ratio and Information UniFrac were calculated in R (https://github.com/ruthgrace/R_Scripts/blob/master/UniFrac.r). PERMANOVA on distances between subject groupings was performed through scikit-bio (v.0.5.5) (30). Dimensionality reduction on distances was performed through PCoA via scikit-bio (v.0.5.5). The first three components of each dimensionality reduction were evaluated through KNN classification via scikit-learn (v.0.21.2). To assess the classification accuracy, KNN classification was performed with 10-fold 50:60 cross-validation evaluating area under curve and average precision-recall (APR) prediction accuracy at each fold iteration via scikit-learn (v.0.21.2). The phylogenetic log-ratios were chosen through Empress community plots and calculated with Qurro both through QIIME2 (v.2021.2).

### Data availability.

The software to perform this analysis is available under an open-source license and can be obtained at https://github.com/biocore/gemelli, and all benchmarking code/analysis can be found at https://github.com/cameronmartino/phylo-rclr-benchmarking. The sequences and biom tables for the EMP500, ECAM, and Urbanization data sets can be found on Qiita (https://qiita.ucsd.edu/) (31) under study IDs 13114, 10249, and 10333 and at EBI or BioProject under ERP125879, ERP016173, and ERP107551.

10.1128/msystems.00050-22.1 FIG S1 Comparison of EMP500 shotgun data to simulation. Download FIG S1, PDF file, 1.3 MB. Copyright © 2022 Martino et al. 2022 Martino et al. https://creativecommons.org/licenses/by/4.0/ This content is distributed under the terms of the Creative Commons Attribution 4.0 International license.

10.1128/msystems.00050-22.2 TABLE S1 Comparison of metrics. Download Table S1, XLSX file, 0.01 MB. Copyright © 2022 Martino et al. 2022 Martino et al. https://creativecommons.org/licenses/by/4.0/ This content is distributed under the terms of the Creative Commons Attribution 4.0 International license.

10.1128/msystems.00050-22.3 TABLE S2 Runtime comparison of metrics on case study data. Download Table S2, XLSX file, 0.01 MB. Copyright © 2022 Martino et al. 2022 Martino et al. https://creativecommons.org/licenses/by/4.0/ This content is distributed under the terms of the Creative Commons Attribution 4.0 International license.
