# Supplementary figures and images for: Compositionally Aware Phylogenetic Beta-Diversity Measures Better Resolve Microbiomes Associated with Phenotype
Source: mSystems. 2022 Apr 28;7(3):e00050-22. doi: 10.1128/msystems.00050-22 (PMC9238373; doi:10.1128/msystems.00050-22)

Observed

Features

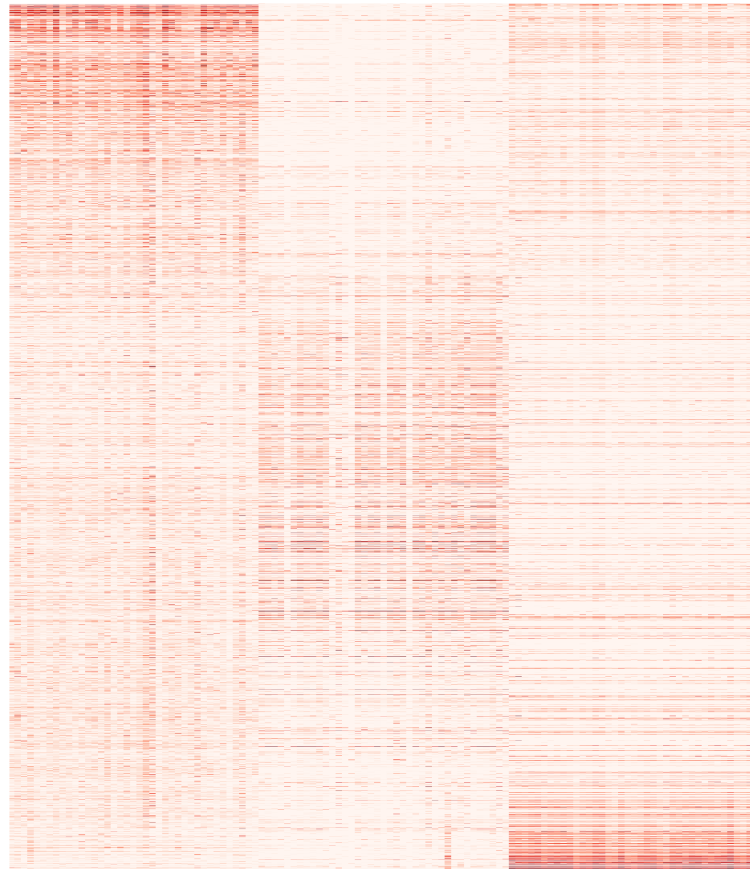

Samples

Simulated

Features

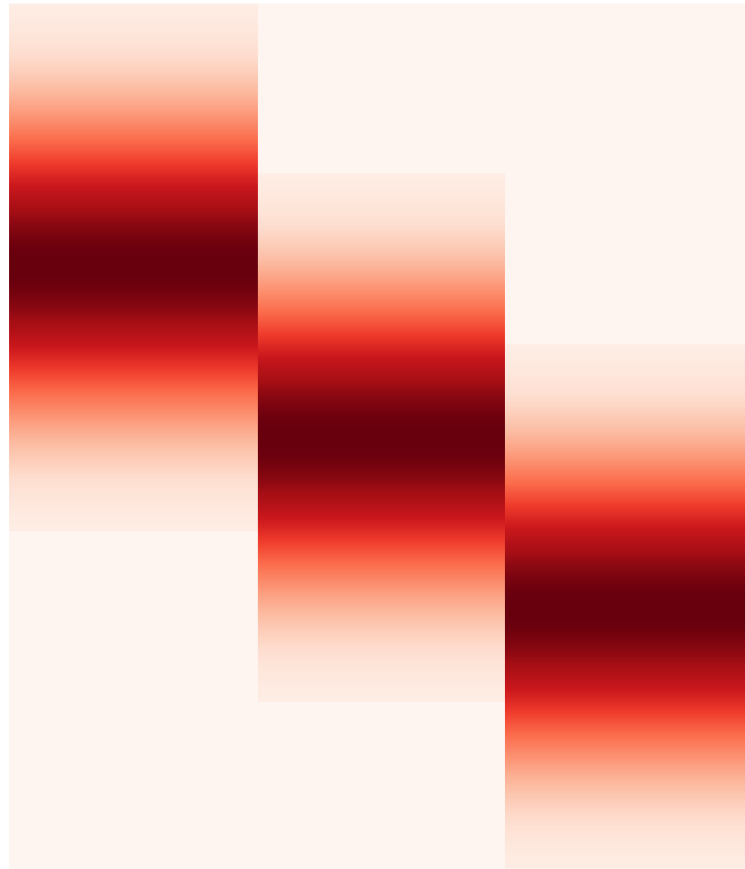

Samples

Simulated

Features

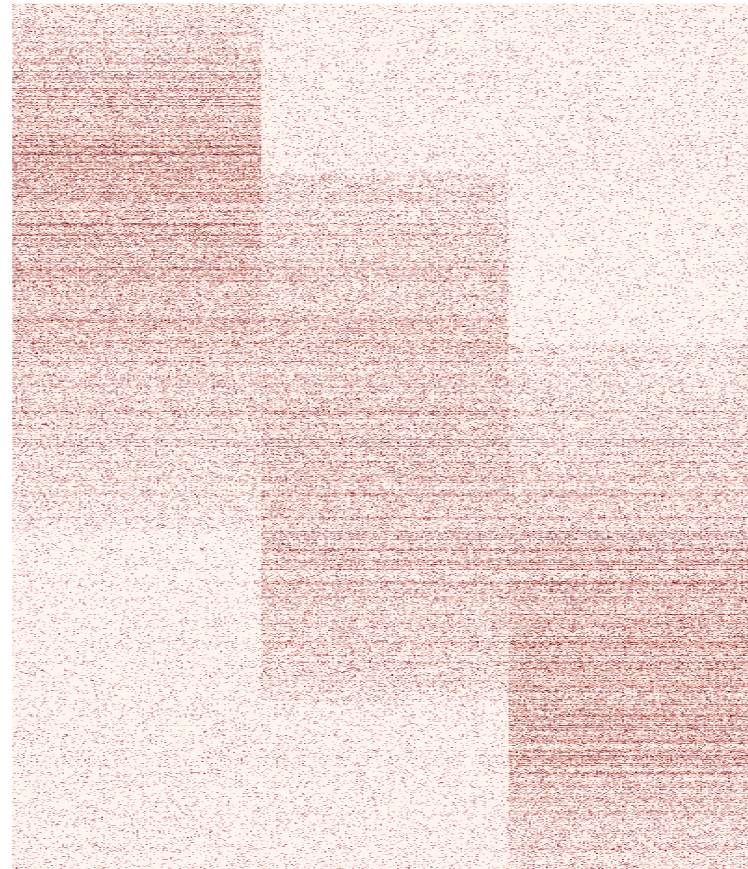

Samples

Supplement: FIG S1 [file msystems.00050-22-s0001.pdf]
